# Role of a non-canonical surface of Rad6 in ubiquitin conjugating activity

**DOI:** 10.1093/nar/gkv845

**Published:** 2015-10-10

**Authors:** Pankaj Kumar, Pearl Magala, Kathryn R. Geiger-Schuller, Ananya Majumdar, Joel R. Tolman, Cynthia Wolberger

**Affiliations:** 1 Department of Biophysics and Biophysical Chemistry, Johns Hopkins University School of Medicine, 725 North Wolfe Street, Baltimore, MD 21205, USA; 2 Department of Chemistry, The Johns Hopkins University, 3400 North Charles Street, Baltimore, MD 21218, USA; 3 Department of Biophysics, The Johns Hopkins University, 3400 North Charles Street, Baltimore, MD 21218, USA; 4 Biomolecular NMR Center, The Johns Hopkins University, Baltimore, MD 21218, USA

## Abstract

Rad6 is a yeast E2 ubiquitin conjugating enzyme that monoubiquitinates histone H2B in conjunction with the E3, Bre1, but can non-specifically modify histones on its own. We determined the crystal structure of a Rad6∼Ub thioester mimic, which revealed a network of interactions in the crystal in which the ubiquitin in one conjugate contacts Rad6 in another. The region of Rad6 contacted is located on the distal face of Rad6 opposite the active site, but differs from the canonical E2 backside that mediates free ubiquitin binding and polyubiquitination activity in other E2 enzymes. We find that free ubiquitin interacts weakly with both non-canonical and canonical backside residues of Rad6 and that mutations of non-canonical residues have deleterious effects on Rad6 activity comparable to those observed to mutations in the canonical E2 backside. The effect of non-canonical backside mutations is similar in the presence and absence of Bre1, indicating that contacts with non-canonical backside residues govern the intrinsic activity of Rad6. Our findings shed light on the determinants of intrinsic Rad6 activity and reveal new ways in which contacts with an E2 backside can regulate ubiquitin conjugating activity.

## INTRODUCTION

Ubiquitination controls a vast array of cellular functions in eukaryotes, including protein degradation, DNA repair, transcription, protein trafficking, the cell cycle and vesicle budding (1–3). Ubiquitin (Ub) is covalently attached to substrate lysine residues through the E1, E2 and E3 enzyme cascade (4–6). In the initial step, the C-terminus of ubiquitin is activated by the ATP-dependent E1 ubiquitin-activating enzyme to form an E1∼Ub thioester complex (4). Ubiquitin is then transferred from the E1 to the active site cysteine of an E2 Ub-conjugating enzyme to form an E2∼Ub thioester (5). In the case of RING E3 ligases, the RING domain binds to both the E2∼Ub thioester and the substrate, stimulating conjugation of the ubiquitin C-terminus to the ϵ-amino group of the target lysine (6). Either a single ubiquitin or one of several types of polyubiquitin chains can be conjugated to a protein, each with a different consequence for the fate or signaling properties of the modified substrate (7). Structural studies have provided snapshots of the different interactions that mediate each step in the ubiquitination cascade (4–6) although our understanding of how differences among E2 enzymes govern substrate specificity remains incomplete.

The nature of the ubiquitin modification is dictated primarily by the E2 enzyme, with important contributions in some cases by the E3 ligase (7). There are ∼35 human and ∼12 yeast E2 enzymes whose polyubiquitinating activity have been studied to varying degrees (8). The majority of E2 enzymes cannot conjugate ubiquitin directly to substrate but depend upon an E3 to position ubiquitin optimally for attack of the substrate lysine on the E2∼Ub thioester (9). In the E2∼Ub intermediate, the flexible C-terminal tail of ubiquitin is tethered by a thioester bond to the active site cysteine but the globular domain of ubiquitin does not adopt a unique position relative to the E2 (10). RING E3 enzymes bind to the E2 and immobilize ubiquitin in a unique position on the E2 that places the ubiquitin-E2 thioester in an optimal position for attack by the ϵ amino group (9,11–13). In this configuration, the globular domain of ubiquitin binds to the same face of the E2 enzyme on which the active site cysteine is located. This positions the ubiquitin C-terminal tail and thioester bond in the appropriate orientation relative to other residues flanking the active site that contribute to the ubiquitin transfer step. The residues in the vicinity of the substrate lysine also play a role in affecting its reactivity and priming it for ubiquitin modification (14–16).

Studies of several E2 enzymes have pointed to a role of the so-called backside of E2 enzymes in governing polyubiquitinating activity (17-21). This face of the E2 enzyme is opposite to that on which the active site cysteine is located and cannot be accessed by a ubiquitin within a single E2∼Ub conjugate due to spatial constraints. However, the backside surface in some cases mediates non-covalent interactions with either free ubiquitin or a ubiquitin in another E2∼Ub conjugate. These interactions were first characterized for UBCH5 isoforms, whose backside mediates self-assembly of UBCH5C∼Ub conjugates and binding to free ubiquitin (17,20,22). The same backside interactions are crucial for the ability of UBCH5C to form a mixture of polyubiquitin chains (17). Backside interactions have also been shown to be important for the ability of other E2 enzymes to form polyubiquitin chains, including human RAD6B (18) and UBE2G2 (23), as well as polySUMO chains in the case of Ubc9 (24). The molecular mechanism by which E2 backside interactions promote polyubiquitin chain formation is unclear, although a recent study (22) suggests that backside interactions may exert an allosteric effect on E2 structure that impacts E2∼Ub reactivity in ways that remain to be elucidated.

Rad6 from the yeast, *Saccharomyces cerevisiae*, and its human homologues, RAD6A/B, are E2 enzymes that play conserved roles in transcription and DNA repair. Rad6 monoubiquitinates histone H2B at K123 in conjunction with the E3 ligase, Bre1, a modification that is important for transcriptional activation and elongation (25–27) as well as mRNA splicing (28). This function is conserved in humans, where RAD6A/B and hBRE1A/B (RNF20/40) play analogous roles (29–31). Rad6 also plays a role in DNA repair, monoubiquitinating PCNA in a reaction catalyzed by the Rad18 E3 ligase (32). Yeast Rad6 and the E3 ligase, Ubr1, also play a role in proteasomal degradation through the N-end rule pathway by attaching K48-linked polyubiquitin to substrates (33,34). In contrast with E2 enzymes that are unable to modify substrates in the absence of an E3, both yeast and human Rad6 homologues can modify substrates on their own. Yeast Rad6 promiscuously polyubiquitinates all four histones in nucleosomes in the absence of an E3 ligase (35) and human RAD6B can form free polyubiquitin chains (18). In the case of the human enzyme, non-covalent back-side interactions are important for the ability of RAD6B to synthesize free chains (18). RAD18 suppresses this activity by competing for binding of ubiquitin to the RAD6B backside, providing further evidence of the importance of these non-covalent interactions to polyubiquitination. The role of the yeast Rad6 backside and its relevance to ubiquitinating activity has not been explored.

To gain molecular insights into the diverse ubiquitination activities of yeast Rad6, we determined the crystal structure of a Rad6∼Ub conjugate at 2.28 Å resolution. The Rad6∼Ub conjugates form a network of interactions in the crystal that are mediated by a non-canonical region of the Rad6 backside that has not previously been observed to participate in self-association of E2∼Ub conjugates. NMR and solution studies support a role for the non-canonical backside in mediating interactions with free ubiquitin and between Rad6∼Ub conjugates. Mutations in the non-canonical backside region dramatically reduce the intrinsic polyubiquitinating activity of Rad6 to a degree comparable to the effect of a mutation in the canonical backside. The effect of non-canonical backside mutations are observed in both the presence and absence of the Bre1 E3 ligase, further pointing to an effect on intrinsic Rad6 function rather than on its ability to be stimulated by Bre1. Our studies shed new light on the role of the Rad6 non-canonical backside in polyubiquitin chain formation while raising new questions about the general mechanism by which ubiquitin binding to the E2 backside contributes to ubiquitin conjugation activity.

## MATERIALS AND METHODS

### Cloning and mutagenesis

DNA encoding human ubiquitin was cloned into a pET3a vector. DNA encoding Rad6 from the yeast, *Saccharomyces cerevisiae*, was cloned into a pMALc2 vector containing an N-terminal maltose binding protein-hexahistidine (MBP-His_6_) tag followed by a Tobacco Etch Virus (TEV) protease cleavage site. Full length yeast Bre1 was cloned into a pET32a vector containing an N-terminal thioredoxin-hexahistidine (TRX-His_6_) tag followed by a TEV cleavage site. All Rad6 and ubiquitin mutants were generated using the Q5 site-directed mutagenesis kit (New England Biolabs) and the incorporation of mutations was verified by DNA sequencing.

### Protein expression and purification

Wild type and mutant Rad6 were expressed as MBP-His_6_ N-terminal fusions in *E. coli* Rosetta2 DE3 pLysS cells. Cells were grown in Luria-Bertani (LB) medium at 37°C to an OD_600nm_ of ∼0.8, induced with 0.5 mM isopropyl β-D-1-thiogalactopyranoside (IPTG) and grown overnight at 18°C before harvesting by centrifugation. Cells were lysed by sonication in lysis buffer containing 50 mM Tris, pH 7.5, 300 mM sodium chloride, 1mM phenylmethanesulfonylfluoride (PMSF), 5mM β-mercaptoethanol and then clarified by centrifugation. The supernatant was passed through a 5 ml HisTrap HP (GE Healthcare Life Sciences) nickel affinity column pre-equilibrated with wash buffer containing 50 mM Tris, pH 7.5, 300 mM sodium chloride, 5 mM β-mercaptoethanol, 20 mM imidazole and 0.1 mM PMSF. After washing the column with 25 column volumes of wash buffer, the protein was eluted with a gradient of 1–1000 mM imidazole. The MBP-His_6_ tag was removed from Rad6 by overnight incubation with 2 mg of TEV protease at 4°C while during dialysis against buffer containing 50 mM Tris, pH 7.5, 100 mM sodium chloride, 5 mM ß-mercaptoethanol and 0.1 mM PMSF. Rad6 was then passed over the nickel-affinity column to remove the MBP-His_6_ tag, remaining uncleaved fusion protein and the His_6_-tagged TEV protease. Fractions containing Rad6 were pooled and further purified on a 5 ml HiTrap Q XL (GE Life Sciences) anion-exchange column developed with a gradient of 1–1000 mM NaCl. Rad6 was concentrated up to 19 mg/ml using 10 000 MWCO Vivaspin^®^ 20 concentrator and stored at -80°C.

Full-length yeast Bre1 was expressed as an N-terminal fusion with a cleavable thioredoxin-hexahistidine (TRX-His_6_) tag in *E. coli* Rosetta2 DE3 pLysS cells. Cells were grown in LB medium supplemented with 50 μM ZnCl_2_ at 37°C to an OD_600nm_ of ∼0.6, induced with 0.25 mM IPTG and grown overnight at 18°C before harvesting by centrifugation. Cells were lysed by sonication in lysis buffer containing 50 mM Tris, pH 7.5, 300 mM sodium chloride, 50 μM ZnCl_2_, 1 mM *tris* (2-carboxyethyl)phosphine (TCEP), 1mM PMSF, and 5–10 μM each of leupeptin, aprotinin and pepstatin protease inhibitors. The whole cell lysate was clarified by centrifugation and the supernatant was passed through a 5 ml HisTrap HP nickel affinity column pre-equilibrated with wash buffer containing 50 mM Tris, pH 7.5, 300 mM sodium chloride, 50 μM ZnCl_2_, 1 mM TCEP, 20 mM imidazole and 0.1 mM PMSF. After washing with 25 column volumes of wash buffer, the protein was eluted with a gradient of 1–1000 mM imidazole. The TRX-His_6_ tag was removed from Bre1 by incubation with TEV protease as described above, except that 50 μM ZnCl_2_ was added to the buffer. Bre1 was then passed over a HisTrap HP nickel-affinity column to remove the TRX-His_6_ tag, remaining uncleaved fusion protein, and the His-tagged TEV protease, which were retained on the resin.

Recombinant hexahistidine-tagged human E1 (His_6_-UBE1) (36), wild type ubiquitin (Ub), hexahistidine -tagged ubiquitin (His_6_-Ub), mutant Ub^G75C^, Ub^Q2A^ and Ub^F4A^ (37), ubiquitin aldehyde (27,38) and the SAGA deubiquitinating module (39) were expressed and purified as previously described.

### Preparation of Rad6^iso^∼Ub conjugates

The method for generating the Rad6^iso^∼Ub isopeptide conjugate was adapted from Plechanovova et al. (9) using a mutant Rad6 protein with the active site cysteine, C88, mutated to lysine. Rad6^C88K^ (150 μM) was incubated with 300 μM His_6_-Ub and 4 μM UBE1 at 35°C for 24 h in buffer containing 50 mM Tris pH 10.0, 150 mM NaCl, 5 mM MgCl_2_, 0.8 mM TCEP and 10 mM ATP. The reaction mixture was then passed through a column containing nickel affinity resin. Rad6^iso^∼Ub-His_6_ bound to the resin while free Rad6^C88K^ was in the flow-through. Rad6^iso^∼Ub-His_6_ was then eluted with 300 mM imidazole. The Rad6^iso^∼Ub-His_6_ conjugate was further purified by anion-exchange chromatography using a MonoQ HR 16/10 column (GE Healthcare Life Sciences) developed with a 0.1 to 1 M NaCl gradient. The purified protein was dialyzed against 50 mM Tris, pH 7.5, 150 mM NaCl, concentrated to 11.6 mg/ml and stored at -80°C until use.

### Crystallization of the Rad6^iso^∼Ub conjugate

Crystals were grown using the sitting drop vapor diffusion method at 20°C in 24% PEG 300, 50 mM sodium acetate, pH 5.4. The crystals appeared in 2–3 days and grew to their final size within 3–4 weeks. Crystals were flash-frozen and stored in liquid nitrogen until use.

### Data collection and structure determination

X-ray diffraction data were collected at a wavelength of 1.03 Å at the GM/CA CAT beamline 23-ID-D at the Advanced Photon Source (APS). The diffraction data were recorded with a PILATUS3 detector and processed with HKL3000 (40). The Rad6∼Ub crystal belongs to primitive orthorhombic space group P2_1_2_1_2_1_ with one Rad6∼Ub conjugate in the asymmetric unit. The crystal structure of Rad6∼Ub was solved by the molecular replacement method using the program, PHASER-MR (41), in the PHENIX suite of programs (42). The coordinates of yeast Rad6 (Protein Data Bank (PDB) code: 1AYZ) and human ubiquitin (PDB code: 1UBQ) were used as search models. The initial solution was subject to multiple rounds of crystallographic refinement with phenix.refine from the Phenix suite of programs (43) and rebuilt to fit the electron density with COOT (44). The final model of Rad6∼Ub has an R-factor of 21.0% and R_free_ of 26.5% for all data between 41.1 and 2.28 Å resolution. The final model has 149 residues in Rad6 and 78 residues in Ubiquitin including the two additional N-terminal residues from the plasmid vector. The crystallographic parameters and final refinement statistics are summarized in Table 1. Figures were prepared using PyMOL Molecular Graphics System, Version 1.5.0.4 Schrödinger, LLC.

**Table 1.** Data collection and refinement statistics

**Crystallographic Data**	
**Space group**	P2_1_2_1_2_1_
**Unit cell**	
a/b/c (Å)	33.66/58.4/115.7
α/β/γ (°)	90.0/90.0/90.0
**Data processing statistics**	
Beam line	APS, 23-ID-D
Wavelength (Å)	1.000
Resolution (Å)^a^	50.0–2.28 (2.37–2.28)
Total reflections	20019
Unique reflections	10705
Completeness (%)^a^	97.5 (95.4)
R_merge_ (%)^a^	10.6 (65.5)
1/σ (I)^a^	7.3 (1.2)
**Refinement Statistics**	
Resolution (Å)	41.1–2.28
R_work_ (%)	21.0
R_free_ (%)	26.5
**rms deviations**	
Bond length (Å)	0.01
Bond angle (Å)	1.19
**Ramachandran plot (%)**	
Most Favored	96.41
Additionally allowed	3.59
Disallowed	0.0
**Number of residues**	
Rad6	149
Ubiquitin	76
Water molecules	12
**Average B-values (Å^2^)**	
Rad6	72.3
Ubiquitin	79.4
Water molecules	72.8

^a^Values in parenthesis are for the highest-resolution shell.

### NMR experiments

The ^15^N-TROSY HSQC spectra for the Rad6:Ub titration experiments were acquired on a Bruker Avance 600 MHz spectrometer at 20°C in 25 mM sodium phosphate, pH 7.5 containing 0.5 mM TCEP. The starting concentration of 70%-^2^H,^15^N-Rad6 was 150 μM with ubiquitin added to a final concentration of 200 μM, 400 μM, 600 μM and 800 μM for the respective titration points. Reported chemical shift perturbation (CSP) values were computed based on measured ^1^H and ^15^N shifts according to the formula Δδ = sqrt((Δδ_N_)^2^ + (5.8*Δδ_H_)^2^), where Δδ_N_ and Δδ_H_ are the chemical shift changes in ^15^N and the ^1^H dimensions, respectively. The *K*_d_ of Rad6:Ub binding was estimated by fitting of the measured CSP values to a 1:1 binding model with the *K*_d_ and the maximum CSP (i.e. that expected for 100% binding) for each residue allowed to float.

The E2∼Ub thioester conjugates were generated in the NMR tube starting from a solution of 250 μM 70%-^2^H,^15^N-Rad6 and 750 μM ubiquitin in a 50 mM Tris, pH 7.5 buffer containing 10 mM MgCl_2_, 1 mM TCEP and 4 mM ATP. The conjugation reaction was initiated by addition of 10 μM human E1 enzyme. The thioester formation reaction was monitored by ^15^N-TROSY HSQC NMR on an 800 MHz Agilent NMR spectrometer at 20°C. EDTA and DTT were added at the final concentration of 10 mM and 30 mM, respectively, to recover free Rad6 and Ubiquitin. NMR experiments with labeled Rad6^L56A^ mutant were performed as for the wild-type protein.

### Microscale thermophoresis (MST) binding assays

Purified Ubiquitin^G75C^ was labeled with fluorescein-5-maleimide (Thermo Scientific, Pittsburgh, PA) for 2 h at room temperature in the dark. Labeled protein was purified using a Superdex 75 10/300 column (GE Healthcare Life Sciences) pre-equilibrated with 50 mM Tris buffer, pH 7.5 and 150 mM NaCl. The Fluorescein-5 maleimide labeled ubiquitin concentration was held constant at ∼150 nM, while the concentration of Rad6 was varied between 2540000 and 77.0 nM. After a short incubation, the samples were loaded into MST standard glass capillaries and the MST analysis was performed using a Monolith NT.115 (NanoTemper). Concentrations of Rad6 on the x-axis are plotted in nM.

### Histone H2B ubiquitination assays

Native nucleosomes purified from HeLa cells (BPS Bioscience) were incubated with the yeast SAGA deubiquitinating module (DUBm), a complex containing Ubp8, Sgf11, Sus1 and Sgf73 (45), to remove endogenous ubiquitin. Nucleosomes (1.25 μM) were incubated with 0.2 μM SAGA DUBm in 50 mM Tris pH 7.5, 50 mM NaCl, 5 mM MgCl_2_, 2.5 mM DTT and 5 mM ATP. After 1.5 h of incubation at 30°C, DUB activity was inhibited by addition of 2.0 μM ubiquitin aldehyde following by incubation for an additional 30 min at 30°C. For the ubiquitination reactions, 20 μM ubiquitin and 2 μM Rad6 were added to the HeLa nucleosome reaction mixture pretreated with DUB module. At the indicated time points, samples were denatured in sample buffer containing SDS, unless otherwise stated, and run on Criterion TGX precast Tris/Glycine 4–15% SDS-PAGE gels. The gels were transferred to PVDF membrane using the BioRad Trans-Blot Turbo transfer system and immunoblotted with anti-H2BK120Ub, anti-H2B, anti-Ub antibodies (Cell Signaling Technology, USA). Secondary antibodies with HRP conjugates were used for detection with SuperSignal West Pico Chemiluminescent Substrate.

### Accession Codes

Coordinates and diffraction data have been deposited in the PDB under accession code 4R62. Rad6 NMR assignments have been deposited in the Biological Magnetic Resonance Bank (BMRB) with accession number 25479.

## RESULTS

### Crystal structure of Rad6∼Ub reveals non-canonical interactions between Rad6 and ubiquitin

**Figure 1.**
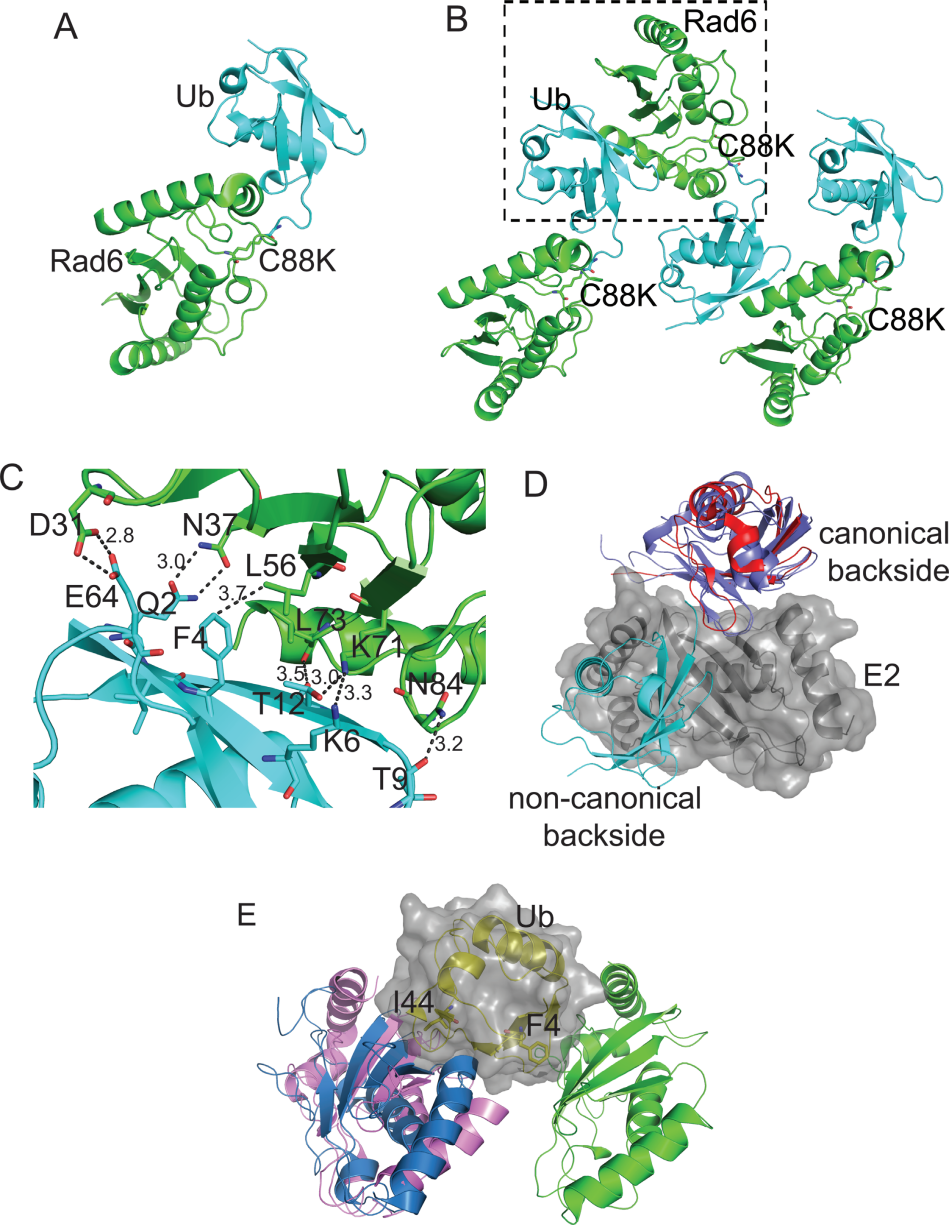
Interactions between Rad6 and ubiquitin in crystals of a Rad6^C88K^∼Ub conjugate. (**A**) Crystal structure of Rad6^C88K^ (green) ∼Ub (cyan). (**B**) Crystal packing interactions formed by Rad6**^C88K^** (green) ∼Ub (cyan) conjugates. Box indicates largest interaction interface. (**C**) Side chain interactions between the ubiquitin (cyan) and Rad6 (green) pair shown in the boxed region of panel B. (**D**) Comparison of non-covalent interactions between E2 enzymes and ubiquitin. The surface of a single E2 (gray) is shown along with the relative position of ubiquitin bound to Rad6 (cyan; this study), Mms2 (blue, PDB ID 2GMI) and UBCH5C (red, PDB ID 2FUH). (**E**) Comparison of non-covalent interactions between ubiquitin and E2 enzymes. The surface of a single Ub (yellow cartoon, gray surface) is shown along with the relative position of Rad6 (green; this study), Mms2 (pale blue, PDB ID 2GMI) and UBCH5C (pale pink, PDB ID 2FUH).

Since the thioester linked Rad6∼Ub conjugate is unstable and thus not suitable for crystallization, we generated a stable Rad6∼Ub conjugate that is not competent for ubiquitin transfer. The Rad6 catalytic residue, C88, was substituted with lysine and ubiquitin was conjugated to Rad6^C88K^ with E1 enzyme, as previously described for UBCH5A (9). The resulting Rad6^C88K^ ∼Ub conjugate contains the ubiquitin C-terminus linked to lysine at position 88 via an isopeptide linkage. The crystal structure of the isopeptide-linked conjugate, which we shall refer to as Rad6^iso^∼Ub, was determined at a resolution of 2.28 Å (Figure 1A and Table 1). In the crystal, Rad6 ^iso^∼Ub conjugates form a network of interactions (Figure 1A and B, Supplementary Figure S1A and S1B). The largest of the interfaces is between Rad6 of one conjugate and ubiquitin in another (Figure 1C and Supplementary Figure S2), which comprises an interaction area of 446.1 Å^2^ as calculated with the PISA server (46). A comparison of the non-covalent interactions between ubiquitin and the E2 backside reveals that ubiquitin interacts with Rad6 in a manner that has not been previously observed (Figure 1D and IE). In previous crystallographic and NMR studies of the E2s, UBCH5C (17,20) and RAD6B (18), as well as the UEV, Mms2 (19), a hydrophobic patch containing ubiquitin residue I44 interacts with the so-called backside region of the E2 which, in Rad6, is centered on residue S25 (corresponding to S22 of UBCH5C (17)). This region of yeast Rad6, however, does not mediate any contacts in the crystal. Instead, the network of ubiquitin-E2 interactions in crystals of Rad6^iso^∼Ub is mediated by a non-canonical backside region of Rad6 centered on residue L56 and a patch on ubiquitin centered on F4 (Figure 1C and E).

### Mutations across the non-canonical backside of Rad6 decrease the polyubiquitination of histone H2B

**Figure 2.**
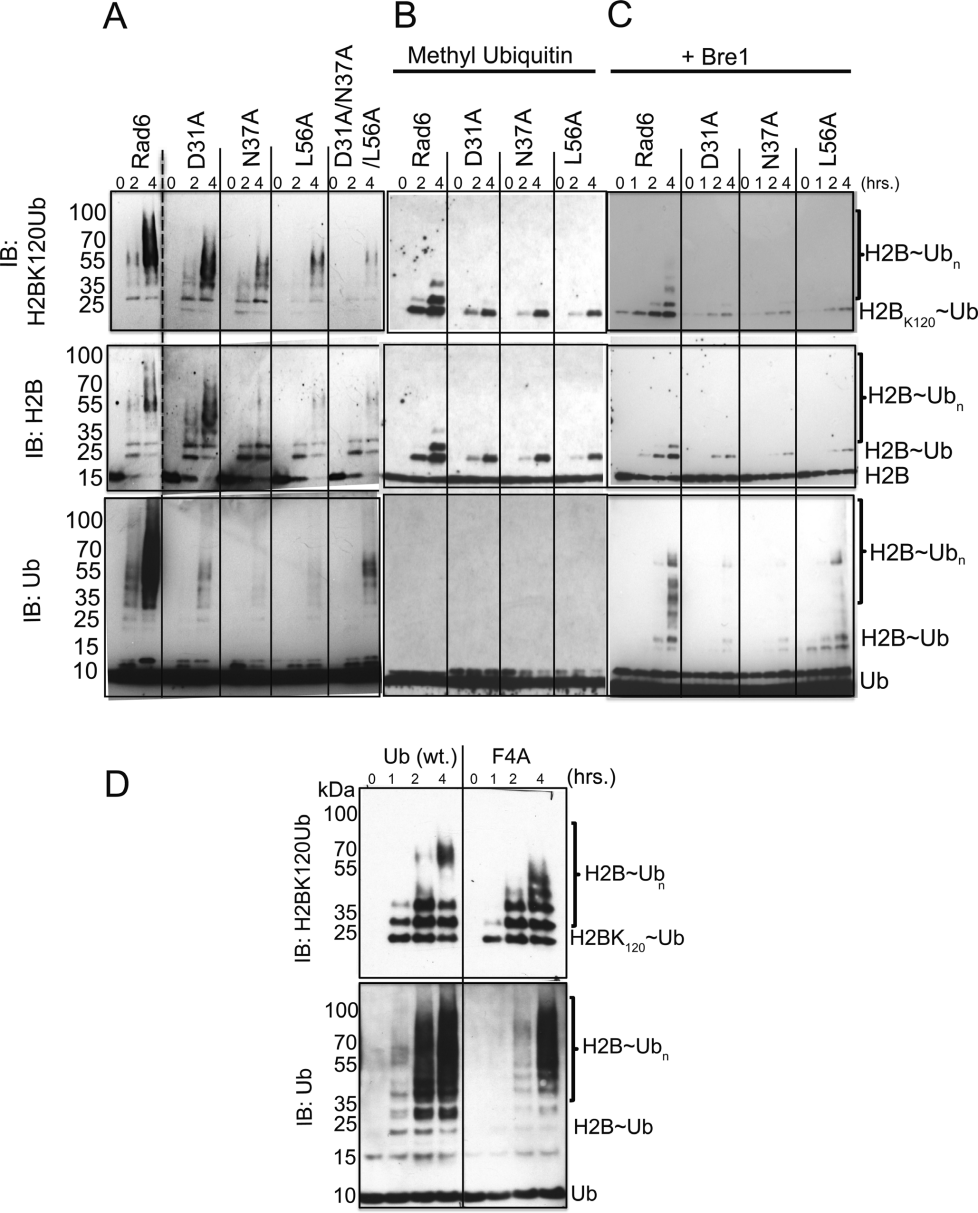
Mutations across the non-canonical backside of Rad6 and F4 hydrophobic patch of ubiquitin decrease ubiquitin conjugating activity. (**A**) Ubiquitination of histone H2B in HeLa nucleosomes by wild-type Rad6 and non-canonical backside mutants Rad6^D31A^, Rad6^N37A^ and Rad6^L56A^. The ubiquitination reaction was performed at 30°C in different time points with 2 μM E2 in the reaction buffer containing 0.1 μM E1, 20 μM ubiquitin, 1.25 μM HeLa nucleosomes. Histone H2BK120Ub, H2B and ubiquitin were probed by western blotting using the anti-H2BK120Ub, anti-H2B, anti-Ub antibodies. Dashed line indicates lanes cropped from right side of gel. (**B**) As in panel A, but with methylated ubiquitin. (**C**) As panel (**A**) with the addition of 10 μM Bre1 E3 ligase. (**D**) Ubiquitination of histone H2B on HeLa nucleosomes with by wild-type Rad6 with Ub (wt.) and Ub^F4A^ mutant.

Non-covalent interactions between ubiquitin and the canonical backside of the E2 enzyme have been observed both in solution and in crystal structures (10,19,20,47) and have previously been linked to the ability of E2 enzymes to form polyubiquitin chains (17). We therefore tested whether the non-canonical Ub-Rad6 interaction observed in the crystal structure of the isopeptide-linked Rad6∼Ub conjugate similarly contributes to Rad6 activity. Since Rad6 can modify nucleosomal substrates in the absence of E3 ligase (35), we tested the effect on H2B ubiquitination of mutations at Rad6 residues D31, N37 and L56, which lie at the largest interface observed in the crystal between Rad6 and ubiquitin (Figure 1C and Supplementary Figure S2). HeLa nucleosomes that were pretreated to remove ubiquitin were used as a substrate in order to take advantage of the availability of antibodies against human H2B monoubiquitinated at K120 (corresponding to yeast H2B-K123) (see Methods). As shown in Figure 2A, mutations D31A, N37A and L56A significantly decrease polyubiquitination of nucleosomes. Rad6 triple mutant D31A/N37A/L56A also shows a marked decrease in histone H2B polyubiquitination (Figure 2A). These mutations do not affect the charging of Rad6 with ubiquitin by E1 (Supplementary Figure S3), indicating that the non-canonical backside mutations disrupt the ubiquitin transfer step and not the ability of Rad6 to form a thioester bond with ubiquitin. We confirmed that the higher-molecular weight species were not due to substrate-independent synthesis of free polyubiquitin chains by assaying Rad6 activity in the presence and absence of nucleosomes (Supplementary Figure S4). Whereas Rad6 alone generates trace amounts of polyubiquitin that is visible after 4 h, polyubiquitination by Rad6 is far more robust in the presence of nucleosomes and is apparent at both the 1- and 2-h time points (Supplementary Figure S4).

The observed pattern of polyubiquitination could be due to the attachment of a single polyubiquitin chain, multiple chains, or result from both polyubiquitination and monoubiquitination of multiple lysine residues in histone H2B. To distinguish among these possibilities, we assayed ubiquitination of histone H2B using methylated ubiquitin, whose chemically modified lysines render ubiquitin incapable of forming polyubiquitin chains. As shown in Figure 2B, Rad6 ubiquitinates up to three lysine residues in histone H2B, indicating that the majority of H2B modification is in the form of polyubiquitin chains. Single mutations, D31A, N37A and L56A, in the non-canonical backside of Rad6 resulted in decreased monoubiquitination of H2B (Figure 2B). The effect of the mutations on H2B multimonoubiquitination was roughly in proportion to their effect on polyubiquitinating activity (Figure 2A and B). This suggests that the non-canonical backside residues play comparable roles in polyubiquitination and multimonoubiquitination.

*In vivo*, Rad6 monoubiquitinates histone H2B in conjunction with the E3 ligase, Bre1 (27,29,35), which directs Rad6 activity to specifically modify H2B-K123(yeast)/K120(humans) and suppresses ubiquitination of other histone residues (29,48) through an as-yet unknown mechanism. We therefore asked whether mutations in the non-canonical backside of Rad6 have the same effects on H2B ubiquitination in the presence of Bre1. As shown in Figure 2C, wild type Rad6 in the presence of Bre1 shows a general decrease in global ubiquitination activity but an increase in H2B ubiquitination at K120, as previously reported (35). Single mutations in the non-canonical backside of Rad6 show a decrease in Bre1-catalyzed ubiquitination (Figure 2C) similar to that observed in the absence of Bre1 (Figure 2A and B). These results indicate that the function of the non-canonical backside of Rad6 is independent of E3 ligase function.

To examine the degree to which the non-canonical backside residues are conserved among other E2 enzymes, we performed a BLAST search that identified E2 enzymes from different organisms that shared sequence conservation throughout the E2 backside (Supplementary Figure S5). A multiple sequence alignment of yeast Rad6 with human RAD6B, RAD6A, UBCH5B, UBCH5C UBC13 and UBE2G2 (Supplementary Figure S6) shows that some of the non-canonical residues are conserved in a subset of E2s (in particular D31/N37), but that none of the human E2s contain all three residues found in yeast Rad6. While L56 is not well-conserved, RAD6B and UBE2G2 contain hydrophobic residues at that position (V and I, respectively). Further studies will be needed to explore the role of the non-canonical surface in these enzymes.

While ubiquitin most commonly interacts with E2 enzymes via a hydrophobic patch centered around I44 (9,17,18,22), the interface between the non-canonical backside of Rad6 and ubiquitin in the crystals of Rad6∼Ub is centered on F4 of ubiquitin (Figure 1C and Supplementary Figure S2). To assess the contribution of this ubiquitin residue to Rad6 activity, we assayed ubiquitination activity using ubiquitin with an F4A point mutation. The Ub^F4A^ mutant exhibits a modest decrease in polyubiquitination of histone H2B (Figure 2D and Supplementary Figure S7) but has a much more pronounced effect on overall polyubiquitin chain synthesis in the presence of nucleosomes (lower panel of Figure 2D). We also assayed the effect of mutating ubiquitin residue Q2, which hydrogen bonds with Rad6 residue N37 at the non-canonical interface. This mutation has a modest impact on ubiquitination (Supplementary Figure S7), suggesting that ubiquitin residue F4 plays the more dominant role in mediating effects at the non-canonical interface. Neither ubiquitin mutation has an effect on E2 charging (Supplementary Figure S8), confirming that the Ub^Q2A^ and Ub^F4A^ mutations impact subsequent steps in ubiquitin transfer. Recent structural and NMR studies show that, in the E2∼Ub thioester poised for ubiquitin transfer, F4 of ubiquitin is exposed to solvent and does not contact either the E2 or the E3 RING domain (9,12,13,17). This mutation is thus not expected to interfere with any of the previously characterized steps of ubiquitin transfer, including formation of a closed E2∼Ub conjugate that is poised for attack by the substrate lysine (9–12).

### NMR detection of interactions between Rad6 and Ubiquitin

**Figure 3.**
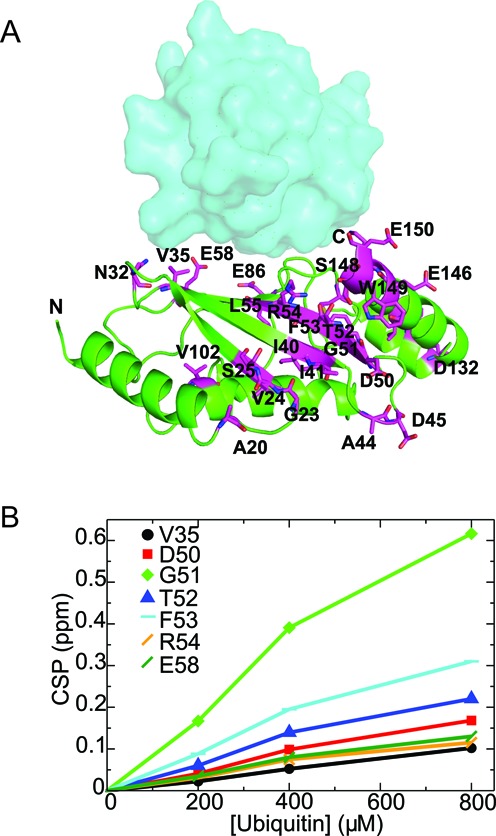
NMR detection of interactions between Rad6 and ubiquitin. (**A**) Mapping of perturbed residues of Rad6 upon binding to free ubiquitin in NMR. The perturbed residues with CSP 0.05 ppm and above are shown in magenta color. Ub (cyan) is shown as surface representation on the non-canonical backside of Rad6. (**B**) Ubiquitin binds to Rad6 with a *K*_d_ above 1 mM. The titrations curves for some of the ^15^N-Rad6 residues that show significant chemical shift perturbations upon free ubiquitin interactions are shown here. The dissociation constant (*K*_d_) was estimated to be ∼1–2 mM assuming a 1:1 stoichiometry and is higher for multiple-site binding models.

The effects of mutating the non-canonical backside of Rad6 support the importance of this surface to ubiquitinating activity but do not directly address whether this surface mediates non-covalent interactions. To test whether the interactions between ubiquitin and Rad6 observed in crystals of the Rad6^iso^∼Ub conjugate occur in solution, we monitored the interactions between free Rad6 and ubiquitin using nuclear magnetic resonance (NMR). We titrated unlabeled ubiquitin into ^15^N-labeled Rad6 and monitored the chemical shift changes in Rad6 spectra as a function of increasing concentrations of unlabeled ubiquitin. Rad6 residues were assigned using standard three-dimensional NMR techniques, as shown in the TROSY-HSQC spectrum of 70% ^2^H, ^15^N Rad6 (Supplementary Figure S9). In the NMR experiment, free ubiquitin induces chemical shift perturbations over a broad range of residues that span the non-canonical backside, the canonical backside and C terminal helix of Rad6 (Figure 3A and Supplementary Figure S10). Rad6 and free ubiquitin interact very weakly in the NMR tube. As such, fitting of the CSP data to a 1:1 binding model yields fairly imprecise results yet the *K*_d_ can be estimated to be above 1.0 mM (Figure 3B). Since free ubiquitin appears to interact with Rad6 at more than one site, this estimate establishes a lower bound for the *K*_d_. The strength of the interaction observed by NMR agrees with binding as assayed by MST, which indicated that the *K*_d_ is greater than 0.5 mM as evident from the inability to saturate ubiquitin binding to Rad6 under the conditions tested (Supplementary Figure S11). Taken together, these results show that free ubiquitin and Rad6 show a broad range of weak interactions with no readily identifiable region that is preferred by a single ubiquitin molecule (Figure 3A, B and Supplementary Figure S10).

**Figure 4.**
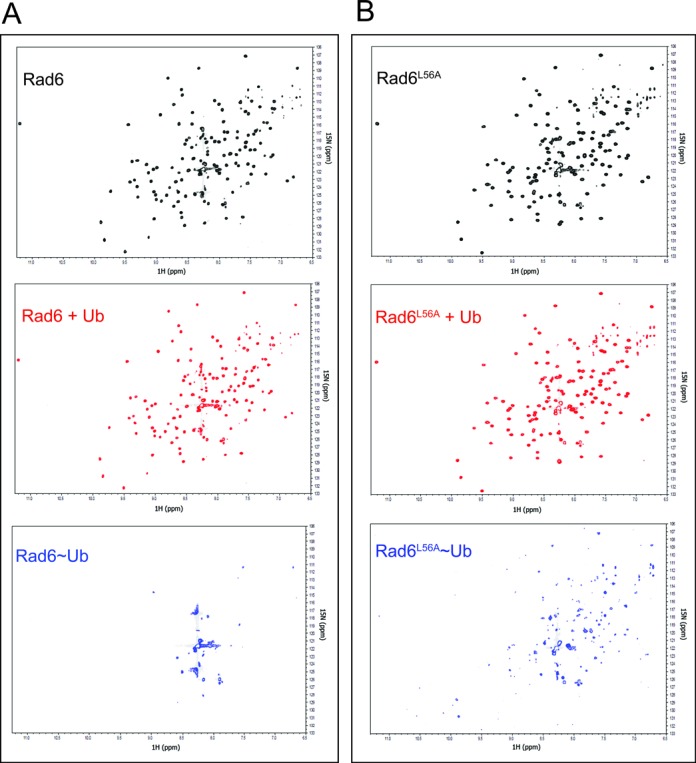
Thioester conjugation reaction of Rad6 and Rad6^L56A^ mutant monitored by NMR. (**A**) Formation of the Rad6∼Ub conjugate shows an NMR spectrum that is indicative of oligomerization. TROSY-HSQC NMR spectra are shown as follows; 250 μM 70%-^2^H,^15^N-Rad6 (black), 250 μM 70%-^2^H,^15^N-Rad6 + 750 μM Ubiquitin (red) and 250 μM 70%-^2^H, ^15^N-Rad6, 750 μM Ubiquitin and 10 μM E1 (blue). (**B**) Oligomerization of mutant Rad6^L56A^∼Ub conjugates as monitored by TROSY-HSQC NMR spectra of ^2^H,^15^N-Rad6^L56A^ and unlabeled ubiquitin as in panel (**A**).

Since the interactions observed in the crystal are not mediated by free ubiquitin, but rather by a ubiquitin conjugated to Rad6, we attempted to replicate this in the NMR tube by monitoring changes in the ^15^N-Rad6 chemical shifts when unlabeled ubiquitin is conjugated to the active site cysteine to form Rad6∼Ub conjugates. However, charging of ^15^N-Rad6 in the NMR tube by addition of E1 enzyme led to disappearance of almost all resonances, which is indicative of greatly reduced molecular tumbling rates (Figure 4A). This behavior is likely the result of self-association of Rad6∼Ub conjugates, but made it impossible to map potential interactions between Rad6∼Ub conjugates. Similar behavior has been observed for UbcH5c, where, formation of UbcH5C∼Ub causes resonances to disappear, indicating self-association of UBCH5C∼Ub conjugates (17). Addition of excess dithiothreitol (DTT) to reduce the E2∼Ub thioester and EDTA to prevent further charging returned the Rad6 ^1^H-^15^N TROSY spectrum to that observed for free Rad6 and ubiquitin (Supplementary Figure S12), confirming that the loss of intensity in the NMR spectrum was directly related to formation of the covalent Rad6∼Ub conjugate species, but not because of addition of E1 interaction with free Rad6.

To test whether interactions between ubiquitin and the non-canonical backside of Rad6 were responsible for self-association of Rad6∼Ub conjugates observed by NMR (Supplementary Figure S12), we assayed the behavior of the Rad6 L56A point mutation (Figure 1C), which significantly decreases Rad6 activity (Figure 2A and Supplementary Figure S2). Charging of ^15^N Rad6^L56A^ with unlabeled ubiquitin in the NMR tube did not have as severe an effect on apparent aggregation activity as judged by the disappearance of far fewer resonance peaks (Figure 4B). This behavior is consistent with less self-association by Rad6^L56A^∼Ub conjugates as compared to ^15^N-Rad6∼Ub (Figure 4A). As in the case of the wild type protein, the NMR spectrum could be regenerated by the addition of reducing agent and EDTA (Supplementary Figure S12). A comparison of the magnitude of the CSP for isotopically labeled wild type Rad6 protein with Rad6^L56A^ in the presence of 750 μM free ubiquitin shows a net decrease in CSPs at most residues (Supplementary Figure S13). The most significant cluster of decreases spans residues 32–55, which encompass the non-canonical backside of Rad6. Taken together, our results support a role for the non-canonical backside of Rad6 in mediating self-association of thioester-linked Rad6∼Ub conjugates (Figure 1B, C and Supplementary Figure S2).

**Figure 5.**
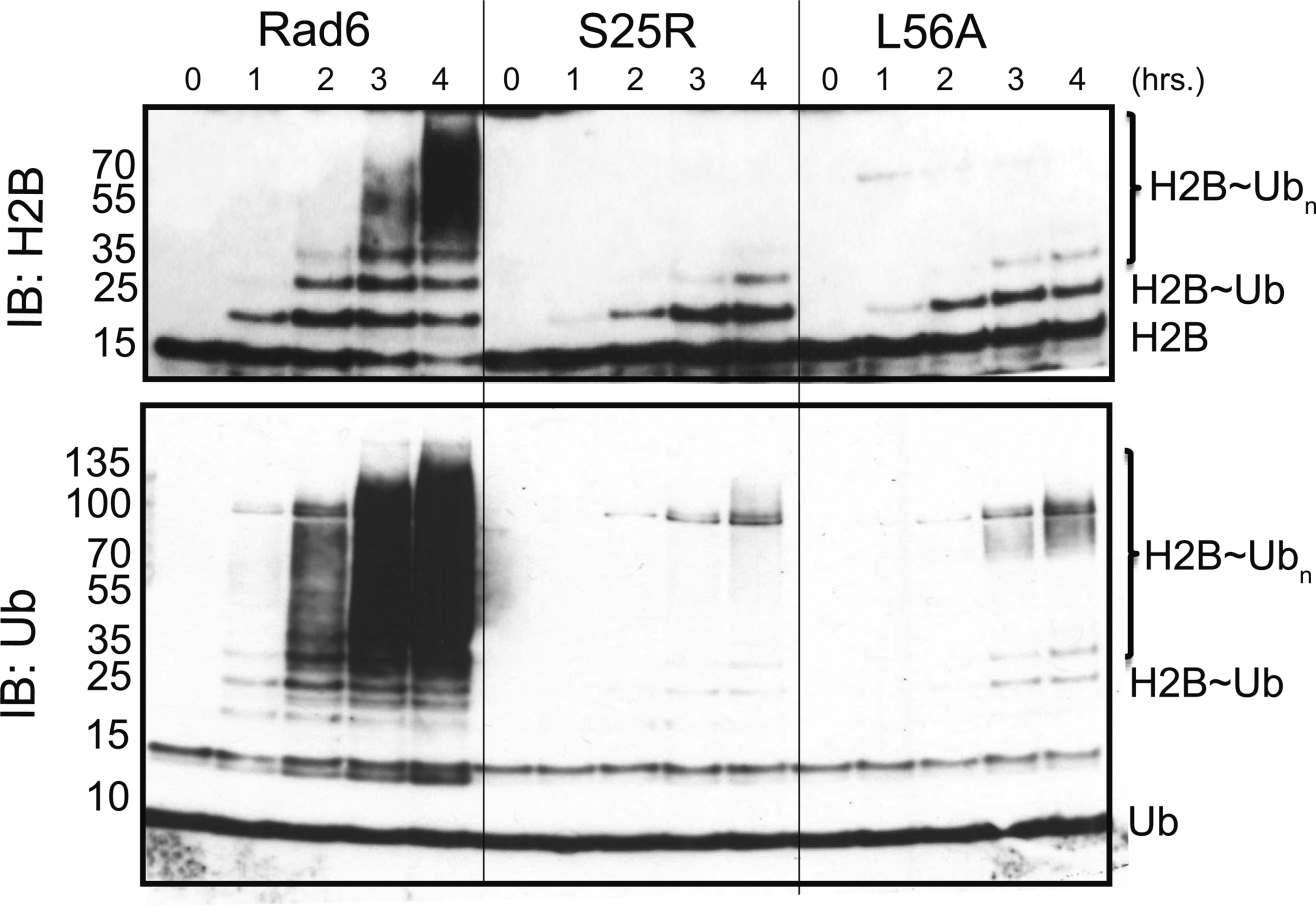
Comparison of the impact of mutations in the canonical and non-canonical backside on Rad6 activity. Ubiquitination of histone H2B in HeLa nucleosomes by wild-type Rad6, canonical backside mutant Rad6^S25R^ and non-canonical backside mutant Rad6^L56A^. The ubiquitination reaction was performed at 30°C at different time points with 2 μM E2 in the reaction buffer containing 0.1 μM E1, 20 μM ubiquitin, 1.25 μM HeLa nucleosomes. H2B and ubiquitin were probed by Western blotting using the anti-H2B, anti-Ub antibodies.

We next asked whether any of the other regions of Rad6 that contact free ubiquitin (Figure 3A) play an important role in Rad6 ubiquitination activity. Since chemical shift perturbations were observed in the canonical backside as well as at other positions on Rad6 in the presence of free ubiquitin (Figure 3A), we mutated selected Rad6 residues in these regions and assayed their role in ubiquitination activity. Residues T52, D132, H133 and E150 were mutated to alanine, while the canonical backside residue, S25, was replaced with arginine to mimic the S22R substitution previously shown to suppress polyubiquitination by human UBCH5C (17). Of these Rad6 mutations, only the canonical backside mutant S25R exhibited a substantial decrease in Rad6 activity (Supplementary Figure S14). The effect of the canonical backside mutation S25R is comparable to that of the non-canonical backside mutant L56A (Figure 5), including dramatic decreases in polyubiquitination and a lesser impact on multi-monoubiquitination. These results indicate that both non-canonical and canonical backside residues play equally important roles in Rad6 ubiquitination activity.

## DISCUSSION

**Figure 6.**
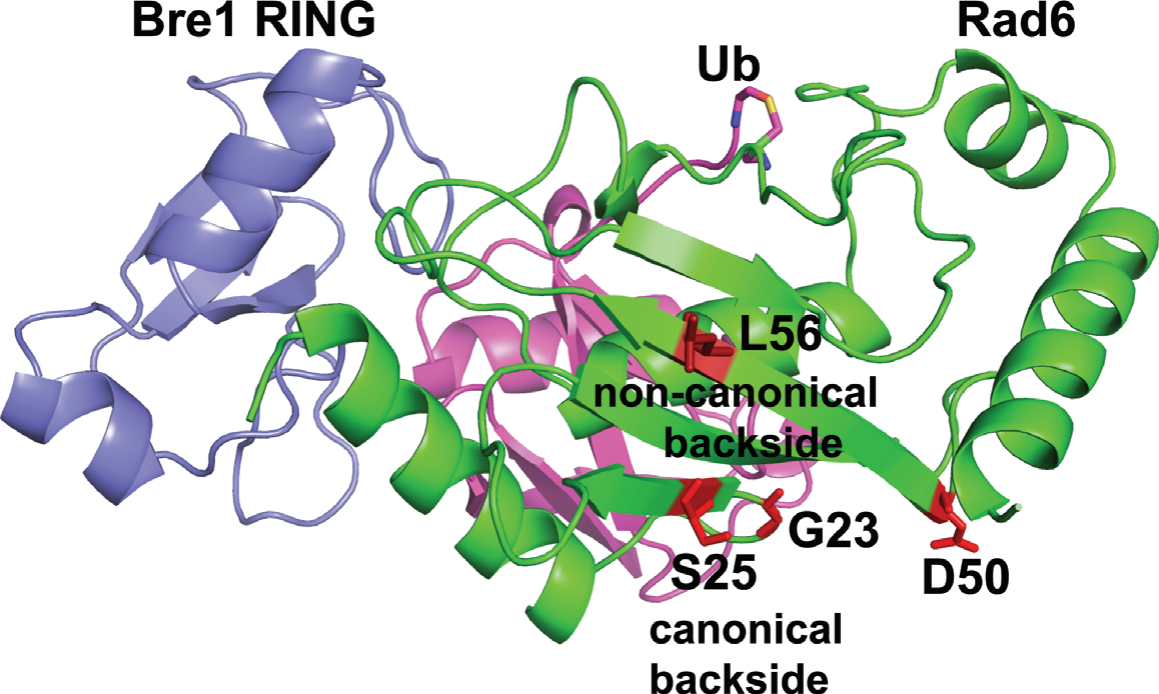
Location of backside and non-canonical backside residues in model of Rad6/Bre1 RING poised for ubiquitin transfer. Model of Bre1 RING domain (blue) bound to Rad6 (green)∼Ub(magenta) conjugate based on the RNF4/UBCH5A∼Ub complex (PDB ID 4AP4). Rad6 non-canonical backside residue L56 and backside residue S25 residue are shown in red stick representations. Residues G23 and D50, which are contacted by the Bre1 RBD domain (48), are indicated in red.

We have identified a new E2 surface that is critically important for Rad6 ubiquitination of histones, and which has both similarities and differences from previously studied backside surfaces (17,18,22,47). The connection between the ability of an E2 to form polyubiquitin chains and the presence of a non-covalent ubiquitin-binding site on the so-called ‘backside’ of the E2 was first discovered for UBCH5C (17,18), which interacts non-covalently with ubiquitin in a region centered on UBCH5C residue S22. Substitution of S22 with arginine was originally reported to selectively decrease polyubiquitination activity (17), although a recent study has shown that the S22R mutation also impacts overall activity of the UBCH5B isoform in both the presence and absence of E3 ligase (22). Structural studies of the canonical backside of human UBCH5B (22), UBCH5C (17,47) and human RAD6B (18) have shown a common non-covalent ubiquitin binding site located on a similar region of the E2 (Figure 1D and E) and that is contacted by hydrophobic residue, I44, of ubiquitin (17,18,22,47). Here we show that yeast Rad6 contains a second site that does not overlap with the canonical backside (Figure 1D), interacts weakly with free ubiquitin (Figure 3 and Supplementary Figure S11), mediates self-association of E2∼Ub conjugates (Figures 1B and 4 and Supplementary Figure S2), is important for both mono- and polyubiquitination of histone H2B in both the presence and absence of E3 ligase (Figure 2) and is situated far from the E2 active site in a location that is inaccessible to the donor ubiquitin linked by a thioester to the E2 active site (Figures 1B and 6). We refer to this surface of Rad6 as the ‘non-canonical backside’ to distinguish it from the previously characterized backside region, which we refer to as the canonical backside (Figure 6). Since non-canonical backside mutations have a comparable effect on yeast Rad6 activity in the presence of the Bre1 E3 ligase (Figure 2C) and have no effect on charging by E1 (Supplementary Figure S3), these Rad6 mutations must be impacting events in the ubiquitin cascade that are intrinsic to the E2 alone. Interestingly, we find that the canonical backside mutation in Rad6, S25R, which corresponds to the previously studied S22R mutation in UBCH5B/C (17,22), also exhibits defects in overall ubiquitination activity that are comparable to non-canonical backside mutations (Figure 5). Together, our data indicate that the non-canonical backside of Rad6 shares many features in common with the well-studied canonical backside, raising the possibility that there are also shared mechanistic features by which interactions with an E2 backside might stimulate ubiquitin transfer.

Although free ubiquitin interacts very weakly with the non-canonical backside, with an estimated *K*_d_ above 1 mM (Figure 3B and Supplementary Figure S11), we observe defects in mono- and polyubiquitination at 20 μM ubiquitin, which is within the expected range of cellular free ubiquitin (49). We speculate that, as in the case of canonical backside interactions with UBCH5B/C, which have a *K*_d_ of about 300 μM, interactions with substrate and the Bre1 E3 ligase may increase the affinity of Rad6 for ubiquitin at either the canonical or non-canonical sites, thus leading to sufficient occupancy of bound ubiquitin to exert an effect on ubiquitin transfer by Rad6.

The role of ubiquitin residue F4 in Rad6 polyubiquitination activity (Figure 2D and Supplementary Figure S7) was unexpected, as most examples of ubiquitin binding, including the binding of free ubiquitin to the canonical E2 backside (17,22), involve I44 of ubiquitin. To our knowledge, the ubiquitin F4 residue does not mediate interactions with E1, E2 or E3 enzymes or with ubiquitin-binding domains (50). For this reason, the effects of mutations of ubiquitin residue F4 have not been well-studied. However, point mutations of ubiquitin F4 and in surrounding residues (Q2, F4, K6, L8 and E64) are known to give rise to defects in endocytosis and cell division in yeast (51,52). The same mutations do not affect proteasomal degradation (52), consistent with a specific effect on particular pathways. A recent study testing the effects of all possible ubiquitin mutations on yeast fitness classified F4 as relatively sensitive to substitution of amino acids other than bulky hydrophobic residues, albeit not as sensitive as I44 substitutions (53). The connection between these observations and our finding that mutation of this ubiquitin residue impacts Rad6 polyubiquitination activity will require further study.

By what mechanism can interactions with either the canonical or non-canonical E2 backside stimulate ubiquitin conjugating activity? It was initially proposed (17) that self-assembly of activated E2∼Ub conjugates facilitate a high local concentration of donor ubiquitin that has the propensity to form polyubiquitin chains. However, a detailed physical model that could explain how conjugated donor ubiquitins could participate in polyubiquitin chain formation is lacking (22). Moreover, a proposed role of E2∼Ub self-assembly in E2 activity fails to account for the dual role of the Rad6 non-canonical backside in both monoubiquitination of histone H2B and polyubiquitin chain formation (Figure 2A and B), nor does it explain how binding of free ubiquitin alone to the canonical backside stimulate ubiquitin discharge from UBCH5B∼Ub conjugates (22). The alternative model, that non-covalent contacts with ubiquitin may in some way allosterically activate the E2 enzyme (13,54), is supported by a recent study of UBCH5B (22) showing that ubiquitin binding to the canonical backside enhances both E3-dependent and -independent E2 activity. While Buetow et al. (22) find that the primary effect of free ubiquitin binding is to increase RING E3 affinity by triggering allosteric changes in helix 1 and the adjacent loop of the E2, they also see RING-independent effects that suggest that allosteric effects also impact intrinsic E2 activity. Whether this effect is transmitted through the core of the E2 to the active site (55) or impacts positioning of the donor ubiquitin (9–11,13,56) remains to be determined.

Several lines of evidence point to a broader role for different types of E2 backside interactions in E2 ubiquitin transfer activity. A number of E3 ligases contain domains outside the canonical RING domain that contact the E2 backside and modulate E2 activity, independent of the effect of the RING (6,18,47,54,57). UBE2G2 is a human E2 enzyme that is allosterically activated by a domain of an E3 ligase called G2BR, which binds to the backside of UBE2G2 and stimulates RING domain binding and ubiquitin transfer (54,57). In the case of human RAD6B and the E3, RAD18, the interaction of the RAD18 domain, R6BD, simply competes with ubiquitin backside binding and thereby suppresses polyubiquitination (18). However, there is evidence suggesting that the Bre1 E3 ligase may allosterically regulate yeast Rad6 through a mechanism more akin to the G2BR/UBE2G2 case (54,57). A recent study (48) showed that binding to the yeast Rad6 backside by the Bre1 RBD (35) independently stimulates ubiquitin discharge from Rad6 (48). In contrast with the effect of the RAD18 R6BD on human RAD6B activity (18), the Bre1 RBD does not simply suppress polyubiquitination by yeast Rad6, as evidenced by the fact that fusing Rad6 to the Bre1 RING domain alone converts Rad6 into an H2B monoubiquitinating enzyme (48). Notably, the location on Rad6 to which the Bre1 RBD binds appears distinct from both the canonical and non-canonical ubiquitin binding sites as judged by the impact of Rad6 mutations, G23R and D50R, on RBD binding (48). Our independent studies thus define a broad surface of Rad6, encompassing the two non-covalent ubiquitin-binding sites and the RBD-binding site, where interactions with the E2 impact reactivity at the active site, which is located on the opposite face of the enzyme (Figure 6). While the mechanism by which binding of either the RBD or ubiquitin to various regions of the E2 backside remains to be elucidated, the analogy with UBE2G2 and UBCH5B suggests that interactions with free ubiquitin, Rad6∼Ub conjugates or Bre1 are most likely to impact Rad6 activity through allosteric effects mediated through the broader beta-sheet backside of Rad6.

## Supplementary Material

SUPPLEMENTARY DATA
