# Supplementary material for: Role of a non-canonical surface of Rad6 in ubiquitin conjugating activity
Source: Nucleic Acids Res. 2015 Oct 10;43(18):9039–50. doi: 10.1093/nar/gkv845 (PMC4605308; doi:10.1093/nar/gkv845)
Supplement: SUPPLEMENTARY DATA [file supp_43_18_9039__index.html]

Role of a non-canonical surface of Rad6 in ubiquitin conjugating activity — Role of a non-canonical surface of Rad6 in ubiquitin conjugating activity — SUPPLEMENTARY DATA 

# Role of a non-canonical surface of Rad6 in ubiquitin conjugating activity

## SUPPLEMENTARY DATA

- SUPPLEMENTARY DATA
