# Supplementary material for: Role of a non-canonical surface of Rad6 in ubiquitin conjugating activity
Source: Nucleic Acids Res. 2015 Oct 10;43(18):9039–50. doi: 10.1093/nar/gkv845 (PMC4605308; doi:10.1093/nar/gkv845)
Supplement: SUPPLEMENTARY DATA [file supp_gkv845_nar-03666-m-2014-File008.pdf]

SUPPLEMENTARY FIGURES

Figure S1

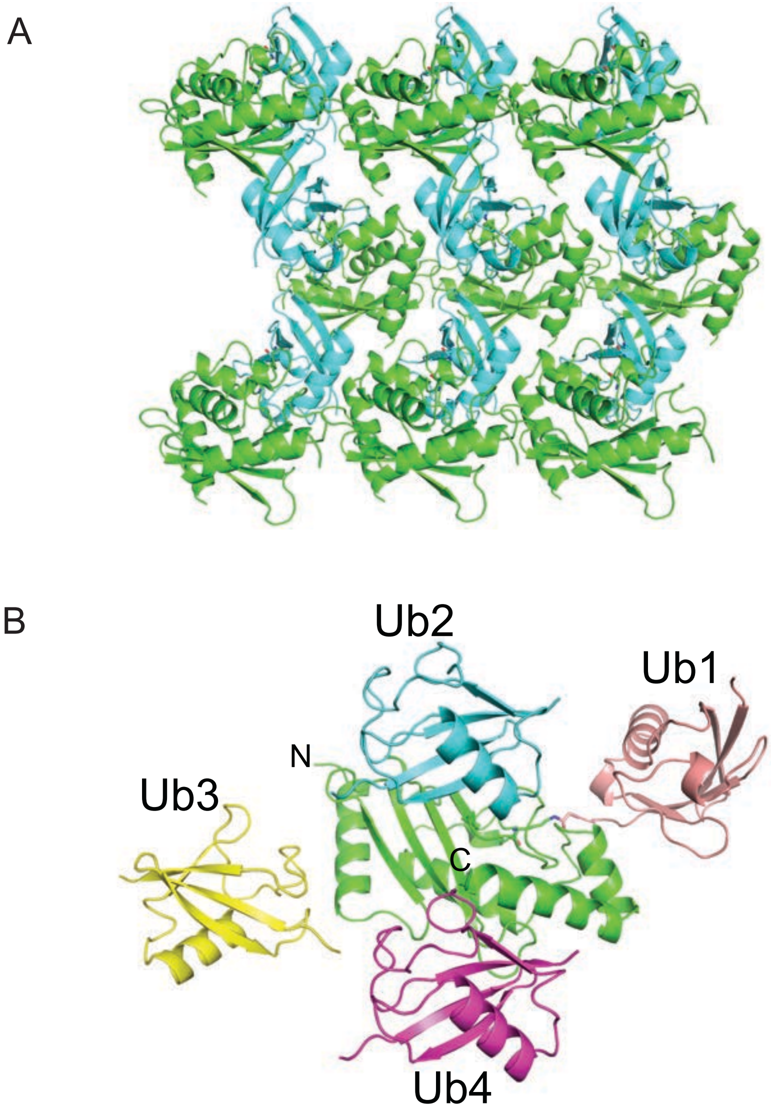

Figure S1. (A) Packing of Rad6~Ub conjugates in the crystal. Rad6 is shown in green and ubiquitin in cyan. (**B**) Packing interactions between Rad6 and four ubiquitin molecules in the Rad6~Ub crystal lattice. The Rad6 (green)~Ub (salmon) conjugate and three symmetry-related ubiquitin molecules that contact Rad6 in the crystal are shown as cartoon representation. Ubiquitin (Ub1) is conjugated to Rad6 via an isopeptide linkage and has a buried interface area with Rad6 of 181.5 $Å^2$. Ubiquitin (Ub2) is involved in interactions through the F4 hydrophobic patch and binds to the non-canonical backside of Rad6 with a buried interface of 446.1 $Å^2$. Ubiquitin (Ub3) interacts across the N-terminal helix of Rad6 with a buried interface of 240.8 $Å^2$. Ubiquitin (Ub4) interacts via its Ile44 surface with Rad6 with a buried interface of 331.9 $Å^2$.

Figure S2

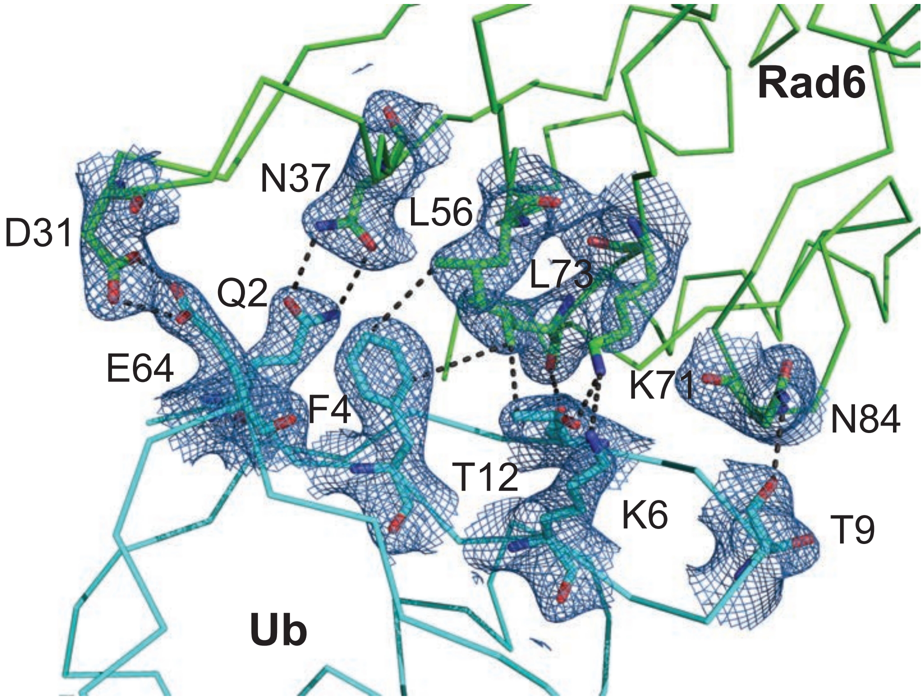

Figure S2. Omit map showing non-canonical backside interactions between Rad6 and ubiquitin in the Rad6~Ub crystal structure. The 2Fo-Fc electron density map contoured at 1.0 σ.

Figure S3

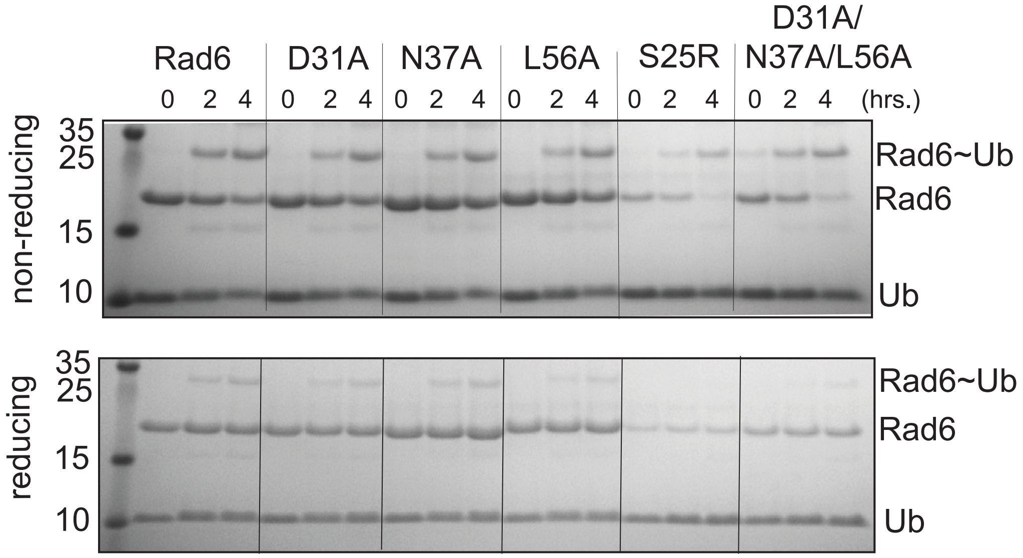

Figure S3. Charging assays for Rad6 mutants. Reactions were performed in 1.0 μM E1, 20 μM Rad6, 50 μM Ub in reaction buffer containing 50 mM Tris, pH 7.5, 150 mM NaCl, and 0.5 μM TCEP at 30˚C. Thioester bond formation was monitored at the indicated time points by SDS-PAGE using sample loading buffer with or without reducing agent as indicated.

Figure S4

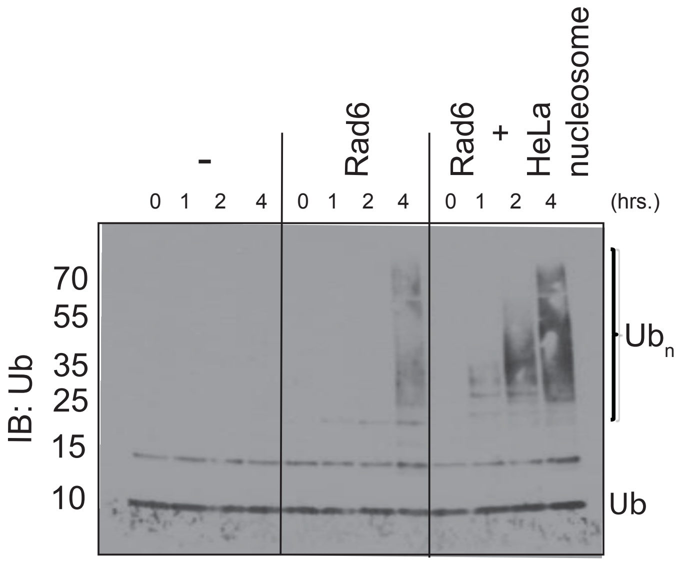

Figure S4. Rad6 ubiquitin conjugating activity in the presence and absence of HeLa nucleosomes. The ubiquitination reaction was performed at 30 ˚C with 2 μM Rad6, 0.1 μM E1, 20 μM ubiquitin, with and without 1.25 μM HeLa nucleosomes. Ubiquitin and ubiquitinated H2B were probed by Western blotting using anti-Ub antibody.

Figure S5

```
                                                                                       D31    N37
RAD6       S.cerevisiae                 ------------------MSTPARRRLMRDFKRMKEDAPPGVSASPLPD-NVMVWNAMII 41
UbcB       Talaromyces stipitatus       ------------------MSTSARRRLMRDFKRMQTDPPAGVSASPVAD-NVMTWNAVII 41
E2 2       Blastomyces dermatitidis     ------------------MSTAARRRLMRDFKRMQTDPPAGVSASPVAD-NVMTWNAVII 41
E2-17kDa   Botrytis cinerea             ------------------MSTAARRRLMRDFKRMQTDPPAGVSASPVAD-NVMLWNAVII 41
E2 2       Trichoderma reesei           ------------------MSTAARRRLMRDFKRMQTDPPAGVSASPIPD-NVMTWNAVII 41
mus-8      Neurospora tetrasperma       ------------------MSTAARRRLMRDFKRMQTDPPAGVSASPVPD-NVMTWNAVII 41
E2 2       Spathaspora passalidarum     ------------------MSTPAKRRLMRDFKRMQQDAPSGVSASPLPD-NVMKWNAVII 41
E2-20kDa   Clavispora lusitaniae        SSLIASKRFLTPHCRNLTMSTPAKRRLMRDFKRMQSDAPSGVSASPVPD-NVMSWNAVII 119
E2 2       Candida tenuis               ------------------MSTPAKRRLMRDFKRMQQDAPSGVSASPLPD-NVMTWNAVII 41
E2 7-like  Musa acuminata               ----------------MAATTQASLLLQKQLRDLMKNPVDGFSAGLVDDSNVFEWNVTII 44
E2 7 iso   Amborella trichopoda         -----------------MEEYRATLLLQKQLKELLKNPVDGFSAGLVDDDNIFEWNVTIT 43
E2 11-like Phoenix dactylifera          ------------MASVYCGGIMANKRIQKELQDLQRDPPTSCSAGPAGE-DLFHWQATIM 47
E2 D2-like Chinchilla lanigera          ---------------------MALQRIQKELADLAQNPPAQFSAGPMGD-DLFVWQATII 38
                                                              *   : :::  :  :.    **.   : ::: *:. *
                                                      L56
RAD6       S.cerevisiae                 GPADTPYEDGTFRLLLEFDEEYPNKPPHVKFLSEMFHPNVYANGEICLDILQN------- 94
UbcB       Talaromyces stipitatus       GPADTPFEDGTFRLVMHFEEQYPNKPPGVKFISQMFHPNVYGTGELCLDILQN------- 94
E2 2       Blastomyces dermatitidis     GPADTPFEDGTFRLVMHFEEQYPNKPPGVKFVSQMFHPNVYGTGELCLDILQN------- 94
E2-17kDa   Botrytis cinerea             GPADTPFEDGTFRLVMTFEEQYPNKPPAVKFISQMFHPNVYATGELCLDILQN------- 94
E2 2       Trichoderma reesei           GPADTPFEDGTFRLVMQFEEQYPNKPPQVKFISQMFHPNVYATGELCLDILQN------- 94
mus-8      Neurospora tetrasperma       GPADTPFEDGTFRLVMHFEEQYPNKPPSVKFISEMFHPNVYATGELCLDILQN------- 94
E2 2       Spathaspora passalidarum     GPSDTPFEDGTFRLILQFDEQYPNKPPSVKFISDMFHPNVYASGELCLDILQN------- 94
E2-20kDa   Clavispora lusitaniae        GPADTPFEDGTFRLVLQFDEQYPNKPPSVKFISEMFHPNVYASGELCLDILQN------- 172
E2 2       Candida tenuis               GPADTPFEDGTFKLVMQFDEQYPNKPPTVKFISEMFHPNVYASGELCLDILQN------- 94
E2 7-like  Musa acuminata               GPPDTLYDGGYFNAIMSFPSNYPNSPPSVRFTSEMWHPNVYSDGRVCISILHPPGDDPNG 104
E2 7 iso   Amborella trichopoda         GPPDTIYEGGCFNAIMSFPYNYPNSPPSVRFTSELWHPNVYPDGRVCISILHSPGDDPNG 103
E2 11-like Phoenix dactylifera          GPAESPYAGGVFFVMIHFPPDYPFKPPKVNFQTKVYHPNINSNGSICLDILKE------- 100
E2 D2-like Chinchilla lanigera          GPNDTPYQGGVFSLIIAFPSSYPIFPPRVTFTTRIFHPNISKHGTICLDILGN------- 91
                                        ** :: : .* *  :: *  .**  ** * * : ::***:   * :*:.**

RAD6       S.cerevisiae                 ------RWTPTYDVASILTSIQSLFNDPNPASPANVEAATLFKDHKSQYVKRVKETVEKS 148
UbcB       Talaromyces stipitatus       ------RWSPTYDVAAILTSIQSLLNDPNTSSPANVEASNLYKDNRREYVKRVRETVEKS 148
E2 2       Blastomyces dermatitidis     ------RWSPTYDVAAILTSIQSLLNDPNTSSPANVEASNLYKDNRREYTKRVRETVEKS 148
E2-17kDa   Botrytis cinerea             ------RWSPTYDVAAVLTSIQSLLNDPNTGSPANVEASNLYKDNRREYTKRVRETVEKS 148
E2 2       Trichoderma reesei           ------RWSPTYDVAAVLTSIQSLLNDPNTGSPANVEASNLYKDNRKEYTKRVRETVERS 148
mus-8      Neurospora tetrasperma       ------RWSPTYDVAAVLTSIQSLLNDPNTGSPANVEASNLYKDNRKEYHKRVRETVEKS 148
E2 2       Spathaspora passalidarum     ------RWSPTYDVSSILTSIQSLLNDPNISSPANVEAANLYKDHRSQYVKRVRETVENS 148
E2-20kDa   Clavispora lusitaniae        ------RWSPTYDVSSILTSIQSLLNDPNISSPANVEAANLYKDHRSQYIKRVRETVERS 226
E2 2       Candida tenuis               ------RWSPTYDVSSILTSIQSLLNDPNISSPANAEAANLYKEHRSQYIKRVRETVENS 148
E2 7-like  Musa acuminata               YELASERWTPVHTVESIVLSIISMLSSPNDESPANVEAAKEWRESRDEFKKKVSRIVRRS 164
E2 7 iso   Amborella trichopoda         YELASERWSPVHTVETILLSIISMLSSPNDESPANIDAAKEWRDHRDEFKRKVSRIVRKS 163
E2 11-like Phoenix dactylifera          ------QWSPALTISKVLLSICSLLTDPNPDDPLVPEIAHIYKNQRSRYEETARAWTQKY 154
E2 D2-like Chinchilla lanigera          ------QWSPALTISMVLLYIYNVLCDPNPNDSFHPEIANLYLKNRAEYERIARRWTQKY 145
                                              :*:*.  :  ::  * .:: .**  ..   : :  : . : .: . .   ...

RAD6       S.cerevisiae                 WEDDMDDMDDDDDDDDDDDDEAD- 172
UbcB       Talaromyces stipitatus       WED--------------------- 151
E2 2       Blastomyces dermatitidis     WED--------------------- 151
E2-17kDa   Botrytis cinerea             WEDN-------------------- 152
E2 2       Trichoderma reesei           WED--------------------- 151
mus-8      Neurospora tetrasperma       WED--------------------- 151
E2 2       Spathaspora passalidarum     WLEDDL---EDDEDDKDDE------ 164
E2-20kDa   Clavispora lusitaniae        WDEDDD---EDGEE--EDE------ 240
E2 2       Candida tenuis               WNDDDDDDEEDDEDEDEDEDDANDK 173
E2 7-like  Musa acuminata               QEML-------------------- 168
E2 7 iso   Amborella trichopoda         QEAL-------------------- 167
E2 11-like Phoenix dactylifera          AMG--------------------- 157
E2 D2-like Chinchilla lanigera          AM---------------------- 147
```

Figure S5. Multiple sequence alignment showing top twelve results of Blast search against the yeast Rad6 sequence.

Figure S6

```
                                                 D31    N37
yRad6     --MSTPA--RRRLMRDFKRMKEDAPPGVSASPL-PDNVMVWNAMIIGPADTPYEDGTFRL
hRad6b    --MSTPA--RRRLMRDFKRLQEDPPVGVSGAPS-ENNIMQWNAVIFGPEGTPFEDGTFKL
hRad6a    --------------------------------------MVWNAVIFGPEGTPFEDGTFKL
UBCH5C    --M---A--LKRINKELSDLARDPPAQCSAGPV-GDDMFHWQATIMGPNDSPYQGGVFFL
UBCH5B    --M---A--LKRIHKELNDLARDPPAQCSAGPV-GDDMFHWQATIMGPNDSPYQGGVFFL
UBC13     --M---AGLPRRIIKETQRLLAEPVPGIKAEPD-ESNARYFHVVIAGPQDSPFEGGTFKL
Ube2g2    GHMAGTA--LKRLMAEYKQLTLNPPEGIVAGPMNEENFFEWEALIMGPEDTCFEFGVFPA

          L56
yRad6     LLEFDEEYPNKPPHVKFLSEMFHPNVYANGEICLDILQ-------------NRWTPTYDV
hRad6b    VIEFSEEYPNKPPTVRFLSKMFHPNVYADGSICLDILQ-------------NRWSPTYDV
hRad6a    TIEFTEEYPNKPPTVRFVSKMFHPNVYADGSICLDILQ-------------NRWSPTYDV
UBCH5C    TIHFPTDYPFKPPKVAFTTRIYHPNINSNGSICLDILR-------------SQWSPALTI
UBCH5B    TIHFPTDYPFKPPKVAFTTRIYHPNINSNGSICLDILR-------------SQWSPALTI
UBC13     ELFLPEEYPMAAPKVRFMTKIYHPNVDKLGRICLDILK-------------DKWSPALQI
Ube2g2    ILSFPLDYPLSPPKMRFTCEMFHPNIYPDGRVCISILHAPGDDPMGYESSAERWSPVQSV

yRad6     ASILTSIQSLFNDPNPASPANVEAATLFKDHKSQYVKRVKETVEKSWEDDMDDMDDDDDD
hRad6b    SSILTSIQSLLDEPNPNSPANSQAAQLYQENKREYEKRVSAIVEQSWNDS----------
hRad6a    SSILTSIQSLLDEPNPNSPANSQAAQLYQENKREYEKRVSAIVEQSWRDC----------
UBCH5C    SKVLLSICSLLCDPNPDDPLVPEIARIYKTDRDKYNR-----ISREWTQK-YAM------
UBCH5B    SKVLLSICSLLCDPNPDDPLVPEIARIYKTDREKYNR-----IAREWTQK-YAM------
UBC13     RTVLLSIQALLSAPNPDDPLANDVAEQWKTNEAQAIE-----TARAWTRL-YAMNNI---
Ube2g2    EKILLSVVSMLAEPNDESGANVDASKMWRDDREQFYK-----IAKQIVQK--SL-GL---

yRad6     DDDDDDDEAD
hRad6b    ----------
hRad6a    ----------
UBCH5C    ----------
UBCH5B    ----------
UBC13     ----------
Ube2g2    ----------
```

Figure S6. Multiple sequence alignment of yeast Rad6 with human RAD6B (UBE2B), RAD6A (UBE2A), UBCH5B (UBE2E2), UBCH5C (UBE2D3) UBC13 (UBE2N) and UBE2G2.

Figure S7

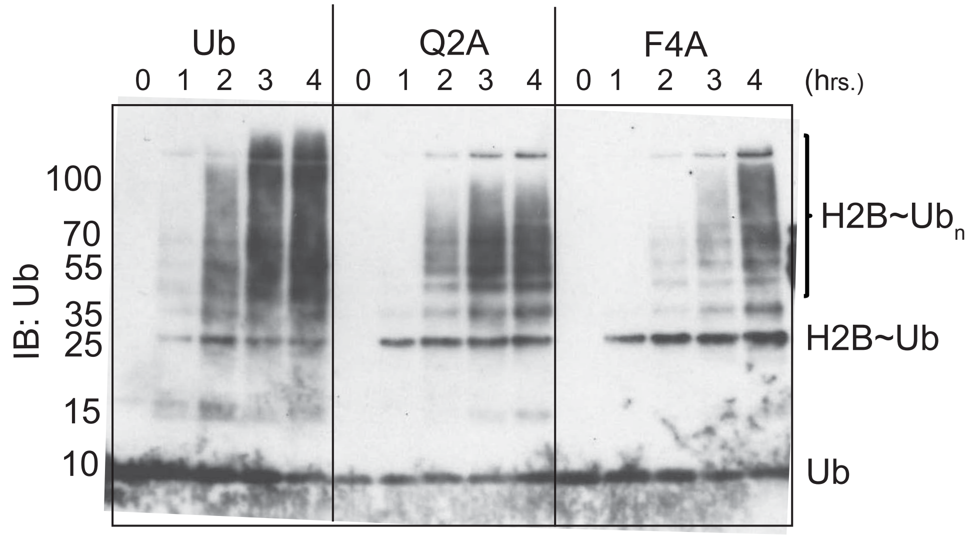

Figure S7. Histone polyubiquitination assays with wild type ubiquitin and mutants Ub^{Q2A} and Ub^{F4A}. The ubiquitination reaction was performed at 30 ˚C at the indicated time points with in reaction buffer containing 2 μM Rad6, 0.1 μM E1, 5 mM ATP, 5 mM $MgCl_2$, 1 mM DTT, 20 μM ubiquitin, 1.25 μM HeLa nucleosomes and 50 mM Tris, pH 7.5. Ubiquitinated H2B and ubiquitin were probed by Western blotting using anti-Ub antibodies.

Figure S8

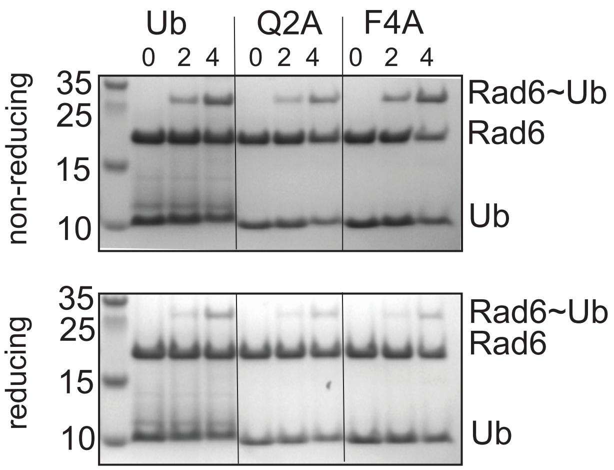

Figure S8. Charging assays for Rad6 with wild-type (Ub) and mutant ubiquitin. Thioester bond formation between Rad6 and either wild type or mutant (Ub^{Q2A} and Ub^{F4A}) ubiquitin was monitored as described in the legend to Figure S3.

Figure S9

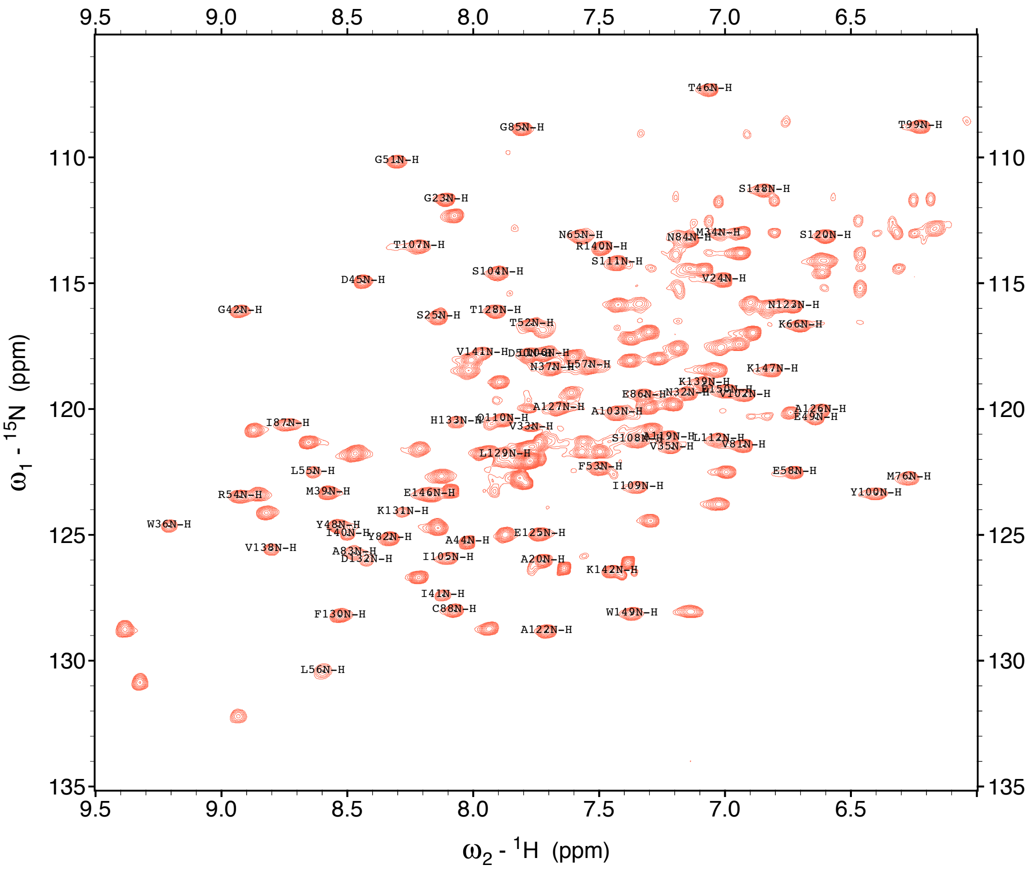

Figure S9. TROSY-HSQC spectra of 70% ^{2}H, ^{15}N Rad6 showing assigned residues.

Figure S10

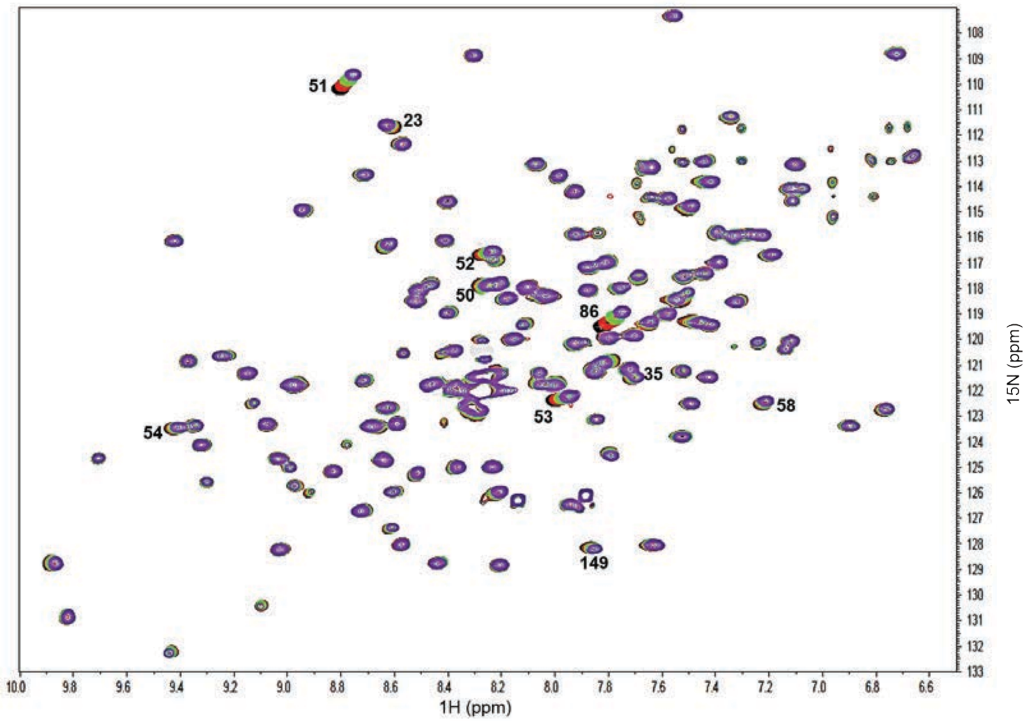

Figure S10. Interaction of Rad6 and ubiquitin as observed by TROSY-HSQC NMR. The starting concentration of 70%-^{2}H,^{15}N-Rad6 was 150 µM, with ubiquitin added to a final concentration of 200 µM (black), 400 µM (red), 600 µM (green) and 800 µM (purple) for the respective titration points.

Figure S11

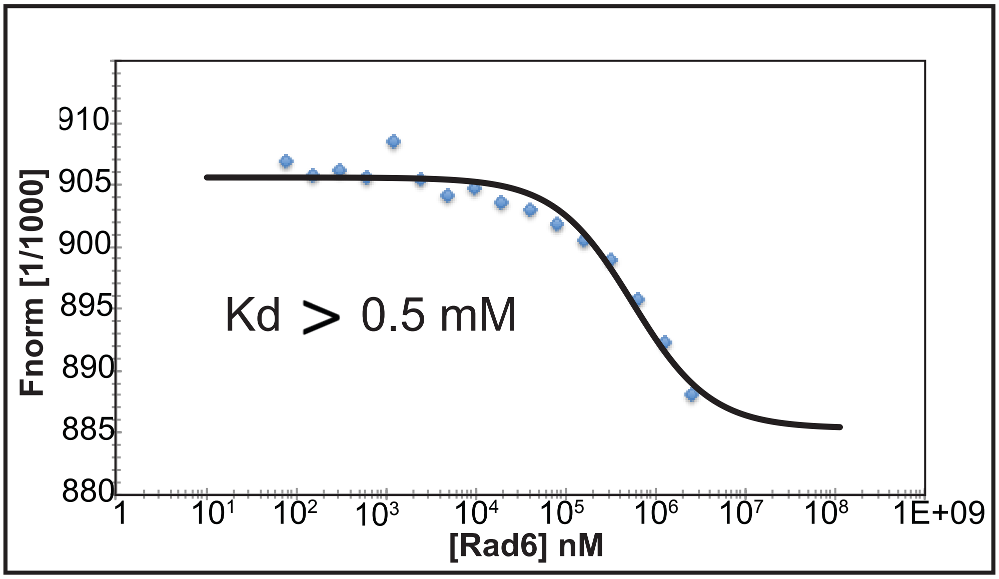

Figure S11. Interaction of Rad6 and ubiquitin monitored by microscale thermophoresis (MST). A titration series of Rad6 with protein concentrations from 2540000 – 77.0 nM was performed while the fluorescein-5 maleimide labeled ubiquitin concentration was held constant at ~150 nM. After a short incubation, the samples were loaded into standard glass capillaries and the MST analysis was performed using a Monolith NT.115 (NanoTemper). Concentrations of Rad6 on the x-axis (blue dots) are plotted in nM. The estimated dissociation constant (K_d) of 561000 +/-26800 nm determined for this interaction assuming a 1:1 binding model constitutes a lower bound, as binding could not be saturated even at the highest Rad6 concentration.

Figure S12

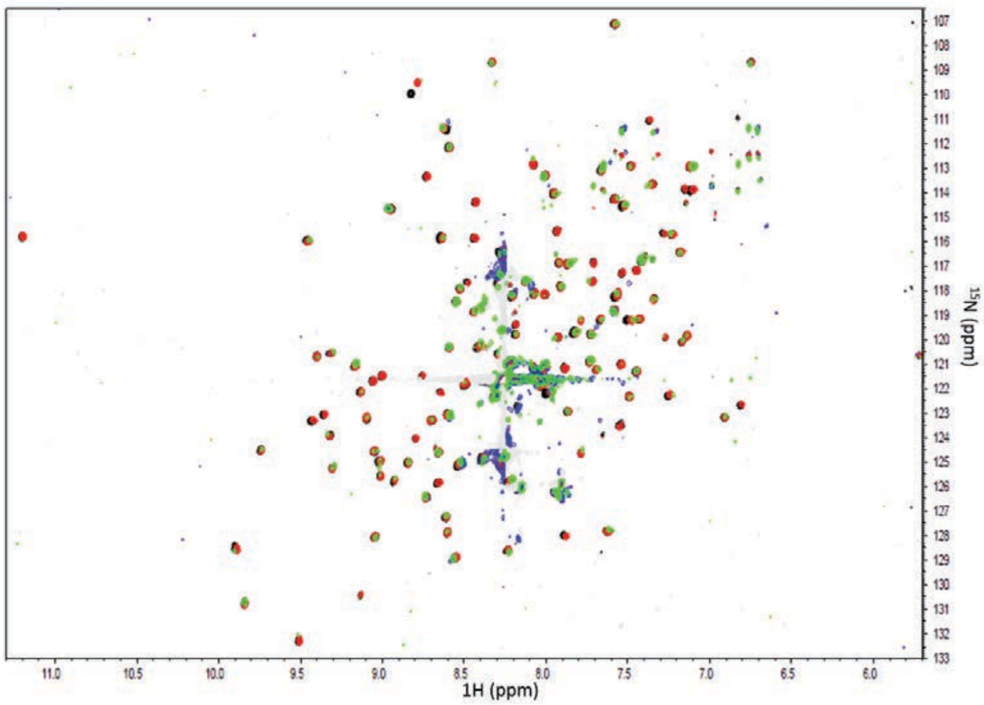

Figure S12. Oligomerization of Rad6~Ub conjugates monitored by NMR and discharging of conjugates upon addition of DTT and EDTA. Shown here are the overlaid [^{15}N, ^{1}H]-TROSY HSQC spectra of 250 μM ^{15}N-Rad6 (black), 250 μM ^{15}N-Rad6 + 750 μM Ubiquitin (red), 250 μM ^{15}N-Rad6 + 750 μM Ubiquitin + 10 mM E1 (blue), 250 μM ^{15}N-Rad6 + 750 μM Ubiquitin + 10 mM E1 + DTT + EDTA (green).

Figure S13

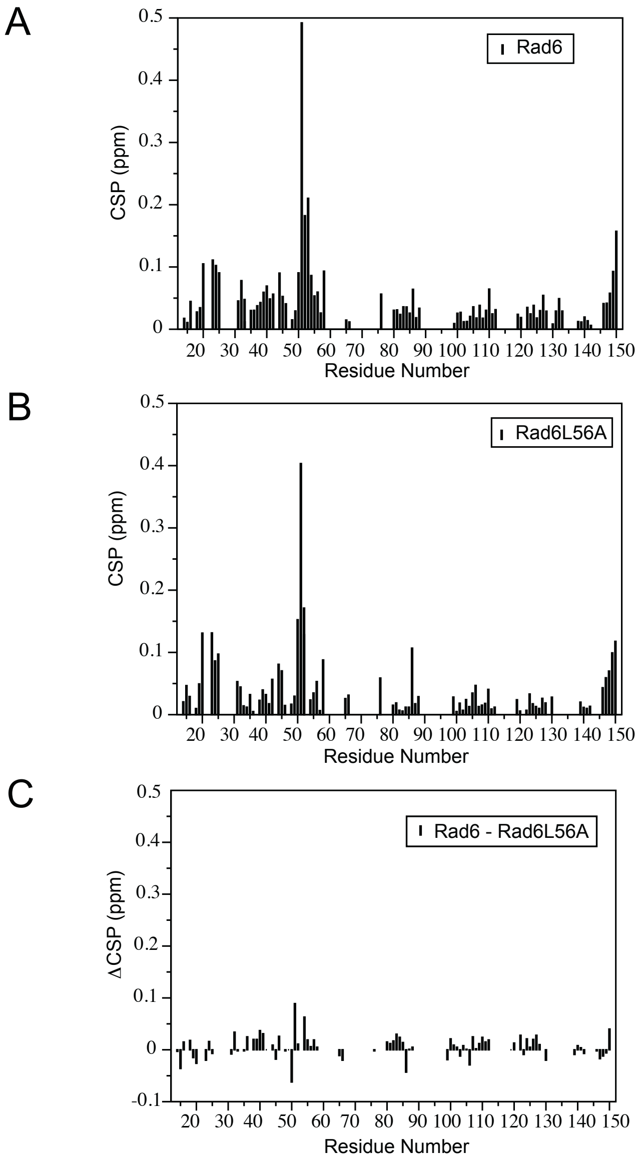

Figure S13. Monitoring of Rad6-ubiquitin interactions by chemical shift perturbation (CSP) (A) CSP data from interaction of 750μM ubiquitin titrated into 250μM ^{15}N-Rad6. (B) CSP data from interaction of 750μM ubiquitin into 250μM ^{15}N-Rad6^{L56A}. (C) CSP difference data of ^{15}N-Rad6 and ^{15}N-Rad6^{L56A} from interaction with ubiquitin.

Figure S14

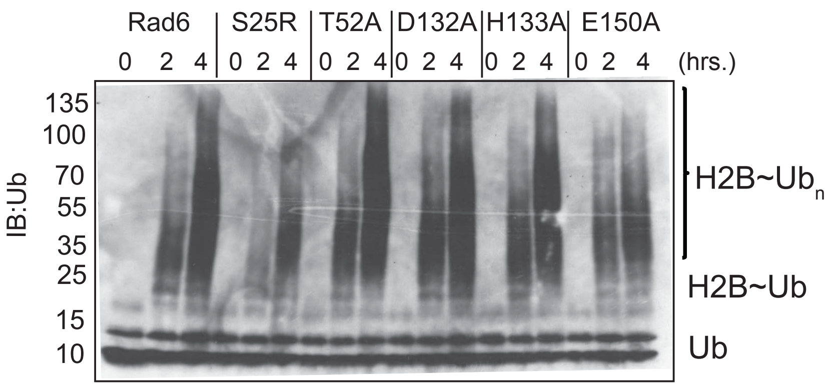

Figure S14. Histone polyubiquitination assays with Rad6 containing point substitutions at residues that show chemical shift perturbation with free ubiquitin. Activity of wild type and Rad6 mutants containing S25R, T52A, D132, H133A and E150A substitutions. The ubiquitination reaction was performed at 30 ˚C at the indicated time points with 2 µM Rad6 in reaction buffer containing 0.1 µM E1, 5 mM ATP, 5 mM $MgCl_2$, 1 mM DTT, 20 µM ubiquitin, 1.25 µM HeLa nucleosome and 50 mM Tris, pH 7.5. Ubiquitinated H2B and ubiquitin were probed by Western blotting using anti-ubiquitin antibodies.
